# Evidence of transfer of miRNAs from the diet to the blood still inconclusive

**DOI:** 10.7717/peerj.9567

**Published:** 2020-09-17

**Authors:** Fermín Mar-Aguilar, Alejandra Arreola-Triana, Daniela Mata-Cardona, Vianey Gonzalez-Villasana, Cristina Rodríguez-Padilla, Diana Reséndez-Pérez

**Affiliations:** 1 Facultad de Ciencias Biológicas, Biología Celular y Genética, Universidad Autónoma de Nuevo León, San Nicolás de los Garza, Nuevo León, Mexico; 2 Facultad de Ciencias Biológicas, Departamento de Inmunología y Virología, Universidad Autónoma de Nuevo León, San Nicolás de los Garza, Nuevo León, Mexico

**Keywords:** XenomiRs, miRNA, Diet

## Abstract

MicroRNAs (miRNAs) are short, non-coding, single-strand RNA molecules that act as regulators of gene expression in plants and animals. In 2012, the first evidence was found that plant miRNAs could enter the bloodstream through the digestive tract. Since then, there has been an ongoing discussion about whether miRNAs from the diet are transferred to blood, accumulate in tissues, and regulate gene expression. Different research groups have tried to replicate these findings, using both plant and animal sources. Here, we review the evidence for and against the transfer of diet-derived miRNAs from plants, meat, milk and exosome and their assimilation and putative molecular regulation role in the consuming organism. Some groups using both miRNAs from plant and animal sources have claimed success, whereas others have not shown transfer. In spite of the biological barriers that may limit miRNA transference, several diet-derived miRNAs can transfer into the circulating system and targets genes for transcription regulation, which adds arguments that miRNAs can be absorbed from the diet and target specific genes by regulating their expression. However, many other studies show that cross-kingdom transfer of exogenous miRNAs appears to be insignificant and not biologically relevant. The main source of controversy in plant studies is the lack of reproducibility of the findings. For meat-derived miRNAs, studies concluded that the miRNAs can survive the cooking process; nevertheless, our evidence shows that the bovine miRNAs are not transferred to human bloodstream. The most important contributions and promising evidence in this controversial field is the transference of milk miRNAs in exosomes and the finding that plant miRNAs in beebread regulate honeybee caste development, and cause similar changes when fed to *Drosophila*. MiRNAs encapsulated in exosomes ensure their stability and resistance in the harsh conditions presented in milk, bloodstream, and gastrointestinaltract to reinforce the idea of transference. Regardless of the model organism, the idea of source of miRNAs, or the approach—bioinformatics or in vivo—the issue of transfer of miRNAs from the diet remains in doubt. Our understanding of the cross-kingdom talk of miRNAs needs more research to study the transfer of “xenomiRs” from different food sources to complement and expand what we know so far regarding the interspecies transfer of miRNAs.

## Introduction

More than 20 years ago, it was discovered that a small RNA, lin-4, played an important role in the development of *C. elegans* (Lee, Feinbaum & Ambros, 1993). At first, it was thought that these novel molecules were exclusive to *C. elegans,* but soon homologues were found in other organisms, including humans (Pasquinelli et al., 2000).

MicroRNAs are 18-to-24-nucleotide-long RNA molecules that are highly conserved among species. They regulate posttranscriptional gene expression either inhibiting mRNA translation or by degrading by exonuclease action, decapping, or deadenylating the poly(A) tail (Fabian & Sonenberg, 2012; Huntzinger & Izaurralde, 2011; Ipsaro & Joshua-Tor, 2015; Jonas & Izaurralde, 2015). In animals, miRNAs are involved in cellular processes such as proliferation, differentiation and apoptosis, as well as in development, metabolism, immune response, and hormone signaling (Alvarez-Garcia & Miska, 2005; Miska, 2005; Bushati & Cohen, 2007; Zhang, Wang & Pan, 2007). In plants, miRNAs are in involved in stress response, homeostasis, and flowering processes (Dugas & Bartel, 2004; Kruszka et al., 2012). Approximately 60% of human protein-coding genes contain at least one conserved miRNA binding site and numerous non-conserved sites (Friedman et al., 2009). Both plant and animal microRNAs have mature lengths ranging from 19 to 24 nucleotides, and both regulate gene expression by recognition of target mRNAs that are often involved in the regulation key developmental events. However, there are also many differences: (a) the first step of animal miRNA biogenesis involves DROSHA nuclease, but this role is carried out by DCL1 in plants; (b) some animal miRNAs are generated from polycistronic transcripts located in intergenic regions of the chromosome, or from introns, whereas the majority of plant miRNAs are derived from single primary transcripts from loci found in the intergenic regions; (c) animal miRNAs mainly act by translational repression using targets at the 3′-UTR, whereas plant miRNAs basically regulate their targets by cleavage in the coding region of the RNA (Zhu et al., 2016).

In 2012, Zhang and cols. suggested that miRNAs from other species (“xenomiRs”), specifically from plants, could withstand the digestion process, enter the animal’s bloodstream through the gastrointestinal tract, and regulate gene expression (Dickinson et al., 2013; Zhang et al., 2012a; Zhang et al., 2012b). After Zhang’s findings were published, different groups tried to replicate the results. Some authors found “xenomiRs” in the blood and tissues of animals and humans, whereas others failed to find any evidence of transfer (Liang et al., 2015; Ma et al., 2017; Masood et al., 2016; Micó et al., 2016; Tosar et al., 2014). It must be noted that the gastrointestinal (GI) tract has special characteristics that must be considered when analyzing whether nucleic acids such as miRNAs can cross this barrier. The GI tract offers a large surface area potentially relevant for cellular uptake and systemic release of miRNAs and other ncRNAs because it contains a large variety of cell types at different maturation levels. However, there are several barriers that could prevent the uptake miRNAs and other ncRNAs by mammalian cells. The first barrier is the oral cavity, where orally ingested RNAs come in contact with lytic enzymes secreted in the saliva. Then there’s the stomach, with its more acidic environment where nucleic acids are denatured and depurinated in the gastric fluid—it is believed that nucleic acids are mostly absorbed as nucleosides. The gastroenteric fluid and peristaltic activity in the GI tract could reduce the contact time between nucleic acids and the epithelial layer, diminishing the opportunities for cellular uptake, or, nuclease enzymes present in the GI tract could degrade nucleic acids before any cellular uptake. Further opportunities for degradation occur in the small and large intestines, where gut flora produces a range of enzymes that degrade miRNAs and other ncRNAs as well. Also, the GI tract is lined by a viscous layer of mucus that traps foreign particles before reaching the underlying epithelium. This layer prevents the access of certain viruses, bacteria, and miRNAs to the underlying plasma membrane by acting as a size-selective diffusional barrier (Dávalos et al., 2019).

To reach the circulatory system miRNAs and other ncRNAs need not only survive cooking and processing, they also need to withstand the conditions in the GI tract. Even then, they would still encounter other biological barriers before exerting any effects. For example, siRNA duplexes are degraded in 15 min when they are incubated in fetal calf or human sera, and siRNA in human plasma is rapidly degraded, with nearly 75% degraded within 2 min. There is relevant nuclease and cleavage activity in plasma and tissues that could degrade RNAs, including miRNAs and other ncRNAs. Extracellular vesicles such as microvesicles, exosomes and apoptotic bodies can offer some protection to miRNAs. Exosomes, for instance, protect their miRNA cargo against degradation and act as a vehicle for cellular uptake of RNAs by endocytosis (Dávalos et al., 2019).

In spite of all these barriers, several research groups in the past eight years have claimed to detect xenomirs in the sera and tissues of different model organisms. At the same time, other groups fail to replicate these findings, even when analyzing the same samples. In this historical review, we describe the research papers that postulate that miRNAs can be transferred from the diet, as well as the ones that postulate that transfer does not exist. We also discuss the findings regarding transfer of exosomal miRNAs from food sources such as milk into the sera and tissues of consuming organisms.

### Survey methodology

Articles reviewed were searched in journal databases. The search terms used included “xenomiRs”, “miRNA”, “crosskingdom”, “miRNAs in diet”, and “transfer”. We also used the key words “plants”, “meat”, “milk” and “exosomes”. We included those articles that directly discussed to the topic of transference of miRNAs from the diet in humans and animal models, and were peer reviewed. We have summarized experiments of miRNAs transference from plants, meat, milk and milk exosomes in Table 1.

**Table 1:** Summary of experimental results on transfer of miRNAs from the diet.

**miRNA source**	**Experimental group**	**Methodology**	**Results**	**Conclusions**	**Authors**	**Year**
Plants: rice	Sera from 11 male and 10 female healthy Chinese, and 8 pooled samples (each pooled from 10 healthy Chinese subjects, mean age)	Deep sequencing and qRT-PCR after oxidation of small RNAs with periodate	miR168a is one of the most highly enriched exogenous plant miRNAs in the sera of Chinese subjects. Functional studies in vitro and in vivo demonstrated that miR168a could bind to LDLRAP1 mRNA, inhibit LDLRAP1 expression in liver.	These findings demonstrate that exogenous plant miRNAs in food can regulate the expression of target genes in mammals.	Zhang et al.	2011
Plants: rice	83 sRNA datasets from tissues of a variety of animals, including humans *Spodoptera frugiperda* and *Helicoverpa zea* neonates	Sequencing analysis and controlled feeding	63/83 datasets had at least one sequenced that matched a known plant miRNA (miR168). 19/83 datasets showed that plant miRNAs were 0.050% of total animal miRNA reads Plant miRNA miR168 was not detected in the tested insects	The appearance of miR168 in datasets does not align with experimental setup. Authors suggest miR168 was the result of contamination from non-plant sources.	Zhang et al.	2012
Plants: Fruit	The plasma and tissues of *Mmu-miR-21*−*/*−** mice, honeybees, and human volunteers were analyzed in search for plant miRNA156a,159a and 169a	Serum analysis	Plant-derived miRNAs were undetectable in human and mice plasma samples. *Mmu-miR-21*−*/*−** Mice expressed negligible levels of miR21 in spite of a diet rich in this miRNA. Only miR156a was detected in bee gut tissue.	Humans are unable to maintain stable levels of plant miRNAs in the bloodstream in spite of diet. Diet-derived miRNAs are ineffective at maintaining miRNA levels in humans, mice and bees.	Snow et al.	2013
Plants: rice	Mice were fed chow containing 75% rice for 1, 3 and 7 days	Controlled feeding study to corroborate Zhang et al. (2011)	Analysis of plasma and liver did not reveal measurable uptake of rice miRNAs	Results did not show uptake of rice miRNAs nor regulation of protein levels in the liver.	Dickinson et al.	2013
Plants	Two macaques were fed a commercially available fruit and protein shake	Feeding experiment	Found extremely low number of copies of plant miRNAs in plasma, or amplification was not specific.	Data does not support that large amounts of plant miRNAs from the diet enter the bloodstream.	Witwer et al.	2013
Plants	Deep-sequencing libraries of small RNAs in a variety of randomly selected studies of human tissues.	Bioinformatics analysis	An experiment was designed to demonstrate that cross contamination during library preparation is the source of exogenous RNA	Contamination is the underlying cause of findings of miRNA transference.	Tosar et al.	2014
Plants	12 raw small RNA sequencing datasets from human and porcine breast milk exosomes.	Bioinformatics analysis	17 plant miRNA species that belong to 11 MIR families were identified in six porcine breast milk exosomes samples, in low abundances.	Plant miRNAs are abundant in exosomes of human and porcine milk, and these molecules have potential target genes in humans	Lukasik et al.	2014
194 species and 5 kingdoms	Analysis of publicly available miRNAs and characterization of features that make them transferable.	Analysis of human circulating microRNAs.	Through a milk feeding experiment, they have validated 9 cow- milk microRNAs in human plasma using microRNA-sequencing analysis.	The data-driven computational analysis is highly promising to study novel molecular characteristics of transportable microRNAs while bypassing the complex mechanistic detail.	Shu et al.	2015
Plants	Mice were fed chow diets supplemented with herbs. Sera and urine were analyzed 7 days after initiating diet.	Controlled feeding experiment	Animals fed herbal diet had increased levels of miR2911. Animals gavage-fed synthetic miR2911 showed increased serum levels.	Small RNAs from plants were delivered into circulation.	Yang et al.	2015
Plants	16 plant miRNAs were measured in the plasma of volunteers who ate fruit or drank fruit juice.	Feeding experiment	miRNA levels increased after eating fruit or drinking juice.	Measured the kinetics of plant miRNAs in human plasma after ingesting watermelon juice.	Liang et al.	2015
Plants: Honeysuckle	Synthetic influenza virus miRNAs were fed to pregnant mice. Mice were gavage fed 0.5 mL of honeysuckle decoction	Gavage feeding study	The level of the influenza virus miRNAs in maternal plasma were elevated significantly. miRR2911 from honeysuckle can be absorbed and delivered into the fetal liver.	Small non-coding RNAs can transfer through the placenta, probably mediated by MVs.	Li et al.	2015
Plants: Olive oil	5 volunteers were fed 40 mL of extra virgin olive oil, and blood samples were taken	Ingestion of extra virgin olive oil	Did not detect olive oil miRNAs in plasma 2 hr after ingestion	Did not detect substantial quantities of olive oil miRNAs in human plasma.	Micó et al.	2016
Pollen	Honeybees were fed a mixture of sugar and miRNA-rich pollen	Feeding experiment	Despite ingesting high levels of miRNAs, bees display systemic levels at concentrations lower than those needed to be functional.	Transfer of miRNAs ingested from the diet is not efficient in honeybees.	Masood et al.	2016
Plants: Maize	Female pigs were fed 1 kg of fresh maize for seven days.	Feeding experiment	Most maize-derived miRNAs could be detected on tissues, and miRNA concentration increased after consuming maize.	Dietary plant miRNAs can enter the blood and tissues through food intake, and mRNAs may be targeted and affected by xenomirs	Luo et al.	2017
Plants: Broccoli	Large nutrigenomics study cohort and in a randomized dose-controlled trial.	Feeding experiment	miRNAs from Brassica olaracea was detected in human subjects that ingested broccoli	Article was Retracted citing loss of confidence in results after a reader pointed incorrect design of miRNA primers.	Pastrello et al.	2017
Plants: Beebread	Rearing of honeybee larvae under laboratory conditions	Bee pollen. the royal jelly, honey, beebread and pollen were obtained in the cole or camellia flowering stage.	miRNAs delay development and decrease body and ovary size in honeybees. One of the miRNAs added to beebread, miR162a, targets mTOR, a gene involved in caste differentiation	Plant miRNAs are able to fine-tune honeybee caste development.	Zhu et al.	2017
Plants: corn	Mice were gavage-fed small RNAs from corn.	Gavage feeding and controlled diet.	There was no difference in miRNA Ct values from liver, blood, fecal and cecal samples among the groups, in both studies.	Corn miRNAs were significantly degraded early in the digestion process.	Huang et al.	2018
Plants	Samples from several authors.	Re-analyzed Lui et al.’s plant mapping results and compared with Ninomiya et al. and Yuan et al., as well as the Sequence Read Archive (SRA).	Only one putative plant miRNA mapped. Several sequences did not map to the species ascribed.	Plant miRNAs probably due to human degradome fragments of plant RNAs, or human miRNAs that have contaminated plant databases.	Witwer	2018
Plants and meat	Fecal and blood samples, biopsies and Foods.	miRNAs analysis in cooked/processed, raw food, mucosa, biopsies and ascites fluid. Diet challenge experiment.	Ultraconserved mammalian miRNAs were present in sufficient quantities in food.	Foods contain large numbers of miRNAs that may be involved in cancer and inflammation. Processing does not affect quantity of miRNAs.	Link et al.	2018
Meat	29 healthy volunteers and divided them in two groups: omnivorous and vegan (control)	Feeding experiment	Beef consumption during a common meal does not promote a significant increase in the levels of miRNAs	A normal beef diet does not result in the horizontal delivery of miRNAs.	Mata-Cardona et al.	2016
Milk	Adults were fed various amounts of milk	Feeding experiment	Meaningful amounts of (miR)-29b and miR-200c were absorbed.	miRNAs absorbed from bovine milk are sufficient to alter human gene expression.	Baier et al.	2014
Milk	—375KO and 200cKO mice	miRNA deficient mice were fed wt murine milk.	No increase in miR-375 levels in enterocytes of jejunum, ileum, colon, and gastric epithelium. No evidence of uptake in plasma, liver, and spleen.	Did not find evidence of uptake in KO mice fed with wt milk in gastric tissues.	Title et al.	2015
Milk	Analysis of publicly available miRNAs and characterization of features that make them transferable.	Analysis, of discriminative features have been used to characterize human circulating microRNAs.	Validated 9 cow- milk microRNAs in human plasma using microRNA-sequencing analysis.	The data-driven computational analysis is highly promising to study novel molecular characteristics of transportable microRNAs.	Shu et al.	2015
Breast milk	Mice pups were fed by wild type or transgenic mice that generates large amounts of miR-30b	Feeding experiment	miR-30b does not increase in pups fed by transgenic vs wild type dams.	No significant differences were observed in tissues and serum of pups fed by transgenic or wild-type dams.	Laubier et al.	2015
Milk	Sera from human donors who participated in Baier et al.’s 2014 milk-consumption study	Analyzed samples from Baier et al. (2014) using Open Array and miRNA qPCR.	Plasma miRNAs did not fill the inclusion criteria and did not seem to be altered by milk consumption.	Found no evidence to support Baier et al.’s conclusion that milk consumption increases levels of bovine miRNAs.	Auerbach et al.	2016
Milk and formula	Analyzed human, goat, bovine and infant formulas to obtain miRNA expression profiles	Next Generation Sequencing and qRT-PCR	Milk contains beneficial miRNAs in the fat portion and encapsulated in exosomes, but these miRNAs were not detected in infant formula. After incubation, miRNAs from milk affected the biological functions of normal and cancer cells.	Some miRNAs from milk may be involved in cell growth and differentiation, and may have a role in the immune system.	Golan-Gerstl et al.	2017
Breastmilk exosomes	4 human and 8 porcine publicly available breast milk exosome sequencing datasets	Mining of datasets and identification of transcripts.	Many miRNAs reported previously come from plants and animals that are not food source	miRNAs in milk are probably artifacts.	Bağci and Almer	2016
Breast milk	Expression profile of miRNAs in human breast milk	qRT-PCR analysis	During the first 6 months of lactation, there was high-expression of immune-related miRNAs. withstand acidic conditions, freeze-thawing, and heat.	Breast milk allows dietary intake of miRNAs. That may act as immune-regulatory agents.	Kosaka et al.	2012
Porcine milk exosomes	Analyzed miRNA expression in exosomes of porcine milk	Deep sequencing analysis	High expression of immune-related miRNAs in calostrum. Resistance to degradation	Exosomal miRNAs could be transferred to the infant gut via breast milk.	Gu et al.	2012
Exosomes from bovine milk	Differentiated and undifferentiated THP-1 cells	THP-1 cells were incubated with labeled exosomes.	miRNAs had higher levels in exosomes than in milk supernatant.	Human macrophages can uptake exosomes from raw bovine milk.	Izumi et al.	2015
Bovine milk exosomes	Evaluation of intestinal transport of bovine milk exosomes using human.	in vitro experiment	Intestinal cells can uptake bovine milk exosomes through endocytosis.	miRNAs are transferred between species through the diet.	Wolf et al.	2015
Milk: exosomes from pork milk	IPEC-J2 cells were incubated with porcine milk exosomes.	Cell proliferation experiment with IPEC-J2 and controlled feeding experiment.	Exosomes from porcine milk promoted proliferation of IPEC-J2 cells.	Milk exosomes regulate cell proliferation and intestinal tract development	Chen et al.	2016
Milk exosomes	Transgenic mice were fed diets with fluorescent-marked exosomes.	Feeding experiment	Fluorescence was higher in liver, spleen and lungs 3 h after gavage feeding, and in liver 24 h after.	Bovine milk exosomes are bioavailable and accumulate in the liver and spleen of mice.	Manca et al.	2018
Various sources	Survey of 823 datasets from human tissue samples	Bioinformatics	miRNAs come from insects or other animals that are not common food sources	Xenomirs have technical rather than dietary origin.	Kang et al.	2017

### Plant- and pollen-derived miRNAs

In 2012, Zhang and cols. found miR168a present in the sera of Chinese subjects, affirming that this miRNA was acquired through the diet. Additionally, the authors found its expression in the liver, and that it reduced the levels of LDL in plasma. They suggested that, in addition to ingesting macro and micro nutrients through the diet, we are ingesting miRNA molecules capable of regulating gene expression (Zhang et al., 2012a).

One year after Zhang’s publication appeared, Dickinson’s group attempted to confirm Zhang’s findings by feeding mice with chow diets with high contents of rice. The results of these experiments do not show apparent uptake of ingested rice miRNAs nor regulation of protein levels in liver and plasma (Dickinson et al., 2013). They did not detect phenotypic changes either, which would be indicative of target gene regulation after rice feeding. Therefore, they did not find evidence of miRNAs transfer from plants to human. Zhang’s group argued that the Dickinson group’s conclusions were the result of deficient methods and missing experiments (Chen, Zen & Zhang, 2013). Dickinson and Zhang’s arguments started the controversy surrounding the possible transfer of diet-derived miRNAs, and ever since, other researchers are working to verify or reject this statement.

Among the first groups to add about miRNA transfer from the diet were Snow et al. (2013), who carried experiments in humans, mice and honeybees to analyze the transfer of miRNAs. They performed controlled feeding experiments on 10 healthy college athletes who ingested fruits containing miR156a, miR159a and miR169a. These plant miRNAs were undetectable in their serum samples, suggesting that humans are unable to maintain the levels of circulating plant miRNAs, despite eating a plant-rich diet. For the mice experiment, Snow and cols. fed the animals with a lard diet containing miR-21, and after four weeks, plasma and organ tissues were collected for analysis. MiR-21 was undetectable in the plasma and organs of *miR-21* −/ − mice, whereas it was abundant in wild-type mice. These data suggest that miR-21-negative mice cannot maintain detectable levels of this miRNA, despite ingesting miR-21-rich food, a result that is similar to the human test described previously. They also analyzed honeybee abdominal tissues and found exceptionally low levels of miR156a, despite pollen being rich in miR156a, miR159a, and miR169a. These results, obtained from experiments in humans and two animal models, suggest that diet alone cannot sustain miRNA levels in the body (Snow et al., 2013).

Soon, another study with contradicting results was published. In 2013, Witwer and cols. fed pigtailed macaques with a commercially available, plant-based, miRNA-rich beverage after overnight fasting. They analyzed the levels of mature plant miRNAs in the macaques’ serum, before the experiment and 1, 4 and 12 h after feeding using RT-qPCR. The authors searched for extremely low copy numbers of plant miRNAs using droplet digital PCR (ddPCR). The intensity plots showed low counts and non-specific amplification of miRNAs. The data from these experiments do not support the hypothesis that miRNAs from plants can be transferred through the diet (Witwer et al., 2013). It is important to consider that detection of plant miRNAs also casts doubt on the specificity and reliability of RT-qPCR, considering that even relatively abundant, consistent miRNAs such as miR160 failed to amplify.

In 2015, Yang and cols. performed a feeding experiment were mice ingested a diet rich in honeysuckle (*Lonicera japonica)*, which contains high levels of miR2911. After seven days of consuming this supplemented chow, they detected differences in the circulating levels of miR2911 in the serum mice offed with the special diet. To confirm, they gavage-fed mice with a single 400-pmol dose of synthetic miR2911, and found that the serum levels of this miRNA doubled 30 m after feeding, but went back to normal after 1 h. Their findings suggest that miR2911 from the diet can be delivered into circulation, albeit for a short time (Yang et al., 2015). Similarly, Li et al. (2015) also found that the transfer of miR2911 from honeysuckle in humans can be delivered to the fetal liver through the placenta, and absorbed. The authors further suggest that these transplacental miRNAs, from the diet or otherwise, can influence fetal development and post-natal health (Li et al., 2015).

The next two studies also contend that miRNAs are transferred from the diet. First, Chen et al. (2016) detected “xenomirs” in mice fed with rapeseed pollen that found 132 clean reads of 34 plant miRNAs, among which miR166a and miR159 were the highest levels in the samples. These miRNAs were present in rapeseed pollen, suggesting that can be absorbed by mice, and circulate with an abundance similar to that of their source (Chen et al., 2016). Then, Masood et al. (2016) evaluated the transference of miRNAs from plants. Bees fed with enriched pollen observed increases of plant miR156a in the midgut after ingestion, but this miRNA were not detected in other tissues, suggesting that miRNAs are not transferred. The authors suggest that the lack of transfer may be due to three factors. First is the limited copies ingested by the honeybees—approximately 6 copies per cell if transport were 100% efficient—which would not reach biologically relevant levels unless special mechanisms were in place. Second, the uptake of pollen-derived miRNAs might change with the insect’s physiological state, such as larval development, and third, gut epithelial tissue might be the most significant barrier for transfer of diet-derived miRNAs, considering that most organisms studied, with the possible exception of *C. elegans* are not competent to absorb dietary miRNAs and transport them into tissues (Masood et al., 2016).

In another controlled feeding study with honeybees performed by Zhu and cols., 16 synthetic plant miRNAs from beebread and pollen were added to the larval diet at the same concentration they are found in nature. The miRNA-enriched beebread delayed development and decreased body and ovary size in honeybees, thus inducing the development of worker bees. The miRNA added to the beebread, miR162a, targets mTOR, a gene involved in caste differentiation (Zhu et al., 2017). The same effect was also observed in *Drosophila*, where plant miRNAs caused extended development times and reduction in fecundity as well as in body and ovary size. These findings are similar to those of Li et al. (2015), who suggested that diet-derived miRNAs can affect fetal—or in this case, larval—development.

In addition, Pastrello et al. (2016) performed a randomized dose-controlled trial in human subjects and found a positive correlation between the amount of broccoli eaten and the amount of miRNAs in the subjects’ blood. They also found evidence that miRNAs from the broccoli are able to regulate the expression of human genes and proteins in vitro. They further suggest that broccoli may have a role in cancer prevention by cooperating with other broccoli-specific compounds (Pastrello et al., 2016). However, a year later, in a letter to Nature, the authors wrote that a reader raised concerns regarding the design of the miRNA primers. They repeated the experiments using the correct primers, but could not amplify the targets in human blood, and thus retracted their paper (Pastrello et al., 2017).

Another pro-transference study came from Luo et al. (2017), who fed fresh maize to pigs for seven days, and detected maize miRNAs in the pigs’ tissues and serum, confirming that maize miRNAs could cross the gastrointestinal tract both in vivo and in vitro. To assess miRNA survival after ingestion, three adult female pigs were fed with fresh maize for seven days, and detected the presence of 16 of the 18 miRNAs in the pigs’ sera and tissues, with relatively low abundance in the pancreas and back muscles. Then, to assess absorption, they used the everted gut sac method to detect higher concentrations of fresh-maize and synthetic miRNAs in the intestinal liquid, suggesting that miRNAs from the diet can cross the intestinal barrier. They also determined that zma-miR164a-5p binds to the porcine target genes *CSPG4*, *OTX1* and *PLAGL2*, contending that this miRNA can be absorbed from the diet and target specific genes (Luo et al., 2017).

Huang and cols. also used corn to study miRNA cross transfer and availability in mice, but their experiment yielded negative results. After the controlled feeding studies, they analyzed whole blood as well as cecal and fecal samples. Corn miR156a, miR164a, and miR167a were not detected in any of the samples and concluded that corn miRNAs are degraded relatively early during digestion and therefore are unable to enter the circulatory system or other tissues. These results show that cross-kingdom transfer of exogenous miRNAs appears to be insignificant and not biologically relevant (Huang, Davis & Wang, 2018).

Similar results were obtained by Kang et al. (2017), who searched for xenomirs in a bioinformatics study of 824 public human sRNA-req databases from serum, plasma, exosomes, and cerebrospinal fluid, as well as from liver and blood cells. The data was processed to eliminate short reads, and then used as control in the feeding study. Rats with a diet of either rice (monocot) or potatoes (dicot) for 28 days; showed neither monocot- nor dicot-specific miRNAs in the rat serum and found instead many rodent-specific miRNAs and one euphyllophyte sequence in one sample. Results concluded that xenomiRs have very low abundance (0.001%) compared to endogenous miRNAs and the tissues and fluids exposed to diet, such as the liver, were not enriched with miRNAs, and that those tissues not exposed to the diet, such as the brain, were not depleted of miRNAs (Kang et al., 2017).

Liu et al. (2017), also performed a bioinformatics study using publicly available datasets from human plasma for the detection of 1301 plant miRNAs, and miR2910 present in all the examined runs, with relatively high abundance. They assumed that if was plant miRNA were so abundant, then it must come from the diet, and then it would be conserved in all kinds of edible plants. Contrary to Kang et al. (2017), their findings suggest that diet-derived miRNAs can transfer into the circulating system. MiR2910 targets human JAK-STAT signaling pathway gene SPRY4 and transcription regulation genes, which adds arguments for the role of diet-derived miRNAs as controlling regulating molecules (Liu et al., 2017).

However, a year later Witwer (2018) re-analyzed Liu and cols. data from their previous year’s study. In the year elapse between both studies, several plant sequences were removed from BMC Genomics because they were actually ncRNA degradation artifacts. Witwer also removed two samples without reliable replicates. After cleaning-up the data, there were 1,294 putative plant miRNAs, although many were paralogs or orthologs, and only a few reads were detected. This time miR159a, miR168a, and miR2911, which were previously described as being highly abundant, were found below threshold level in the plasma samples. In light of the absence of reads, Witwer relaxed the inclusion criteria. Despite this, only 2 of the 15 miRNAs identified by Liu et al. (2017) were annotated in miRBase, and even these two sequences are questioned because one of them maps to mRNA, and the other can be found in many plant transcripts. Witwer cautions against assuming that plant sequences found in human samples come from the diet, and that artifacts and contamination remains the simplest explanation of the findings (Witwer, 2018).

Link and cols conducted one of the most recent and complete studies on miRNA transference (2019). They asked whether different diets affected miRNA concentrations in blood and feces. In their study, human subjects ate a vegetarian diet for 5 to 7 days, and then a meat-rich diet for the same amount of time, then analyzed their blood and feces. The interventions showed no effect on the levels of miR-21 and miR-155, although subjects showed significantly increased levels of miR-168 level in feces after the vegetarian diet. The sera of the subjects, however, did not show differences in the levels of miR-21, miR-155, or miR-16 (Link et al., 2019). Authors detected miR-168 in mucous tissue of the stomach and colon of patients who were fasting overnight, or who have had a colon cleanse before a colonoscopy. They also detected miR-168 in the samples of ascitic fluid of patients with cirrhosis. The tested tissues from normal and cancerous colonic mucosae show presence of miR-168, which was found in higher quantities than in normal tissue. Their findings suggest that plant-derived miRNAs might pass and be absorbed by the mucous membrane of the gastrointestinal tract, and possibly be transferred into the peritoneum (Link et al., 2019).

In summary, some studies of plant- and pollen-derived miRNAs confirm the transfer of diet-derived miRNAs and other do not confirm the transfer. In spite of the biological barriers as well as the mechanisms of cellular uptake that may limit miRNA transference: (i) plant-derived miR-168 was detected in normal tissue, suggesting that it might be absorbed by the mucous membrane of the gastrointestinal tract, and possibly be transferred into the peritoneum. (ii) MiR2911 from the diet can be delivered into circulation, albeit for a short time, (iii) Diet–derived miR162a has found to target mTOR, a gene involved in caste differentiation affecting fetal or larval development. (iv) Several diet-derived miRNAs can transfer into the circulating system and target human and porcine genes for transcription regulation, which adds to the argument that miRNAs can be absorbed from the diet and target specific genes controlling regulating molecules. However, many other studies show that cross-kingdom transfer of exogenous miRNAs appears to be insignificant and not biologically relevant. The main source of controversy in plant studies is the lack of reproducibility of the findings. For example, the first article reporting the possible transfer of miRNAs from rice into the bloodstream was refuted a year later. So far it is not clear if we can consider that there is a transfer of miRNAs from rice to humans. There is also controversy in the area of bioinformatics as well, with studies postulating that miRNAs from the diet are present in low abundance, and others postulating that diet-derived miRNAs are very abundant and even determine the possible target genes. We think that before it can be claimed that plant miRNAs pass into the bloodstream, it is necessary to have irrefutable evidence and reproducible findings.

### Meat-derived miRNAs

There are fewer studies regarding the transfer of miRNAs from meat and other animal products. This may be attributable to the fact that animal and human miRNAs are nearly identical, and thus would not be easy to discriminate in a test. Most studies have focused on whether miRNAs from animal products can survive cooking, curing, drying and other processes.

The first study on meat-derived miRNAs was performed by Dever and cols., who used deep-sequencing and quantitative reverse transcription PCR (RT-qPCR) to characterize the human homologous miRNA profiles in raw, cooked, and pasteurized freeze-dried extracts of bovine-sourced sirloin, heart and adrenal tissue. They found that pan-frying at 177 °C caused a slight reduction in the miRNAs detected in adrenal and heart tissue, but the miRNA profile of cooked sirloin remained unchanged. They also found that flash-pasteurized, freeze-dried tissues retained the miRNA profiles they had when raw. The authors conclude that common food processing techniques such cooking and frying do not change the miRNA profiles of edible beef tissue (Dever et al., 2015). Similarly, Link et al. (2019) analyzed raw and cooked minced pork, raw and well-done beef, charcuterie such as salami, poultry mortadella and liverwurst, whole and separated eggs, smoked salmon, cheese and whole milk. They found that ultra-conserved miRNAs (miR-21, miR-155, and miR-16) are preserved in foods, and are not degraded during cooking or processing, and were present in sufficient quantities. This group also found low concentrations of miRNAs from plants and found differences on miRNA concentration according to the source; the lowest concentration was found in dairy products. They also analyzed miRNA concentration before and after cooking, and found negligible changes (1.5-fold), concluding that miRNAs can withstand the cooking process (Link et al., 2019).

In our laboratory, we performed an experiment to verify whether miRNAs from cooked beef were able to reach the human bloodstream. First, we analyzed the miRNA content of roast beef, reaching a similar conclusion to Dever et al. (2015). Then, we recruited 29 healthy volunteers (14 males and 15 females) and divided them in two groups: omnivorous and vegan (control). We fed the omnivorous group a meal consisting of 200 g roast beef with rice and a lettuce, tomato and lentil salad. The control diet consisted of the same intervention, without the roast beef. We decided to track *hsa*-miR-10b-5p and *hsa*-miR-1-3p because these miRNAs are the most highly expressed in both raw and cooked meat and *hsa*-miR-22-3p has low levels of expression, but was included to determine whether a miRNA with low levels of expression could pass from beef to human plasma (Dever et al., 2015). It is important to point out that the sequences of the human and bovine miRNAs are identical, or present changes in only a single nucleotide, which makes it difficult to specifically quantify beef miRNA by PCR. Therefore, higher levels of a miRNA in human plasma in the omnivorous group would be indicative of transfer from beef. Our results showed that beef consumption during a common meal does not promote a significant increase in the levels of miR-1-3p, miR-10b-5p and miR-22-3p, supporting that miRNAs transfer from diet is unlikely, and therefore, a normal beef diet does not result in the horizontal delivery of miRNAs (Mata-Cardona, 2016).

Contrary to the studies in the previous section, which used raw or unprocessed honey and plant materials for their analysis, the works analyzed in this section used processed animal products. The works reviewed here coincide in that miRNAs can survive processes such as cooking, drying, and pasteurization, which is necessary if these molecules are to perform any regulatory role in humans, however, they make no claims that miRNAs from animal products are transferred from the diet. The fact that animal miRNAs are practically identical to human miRNAs makes their detection and quantification more difficult.

### Milk-derived miRNAs

Milk is an important source of nutrients for mammals that contains growth factors, vitamins, lipids, immune cells, hormones, as well as other bioactive compounds. It also contains large amounts of miRNAs, which begged the question whether they can be transferred, for instance, from mothers to newborns and from bovines to humans, and most importantly if milk can act as a bioactive compounds capable of regulating gene expression. In order to be transferred, miRNAs need to be stable and withstand several unfavorable conditions in milk such as RNases, pasteurization, and barriers in the consumer organism such as RNases, acidic pH in the gastrointestinal tract, digestive enzymes, microbiota, among many others (Benmoussa & Provost, 2019). It has been shown that the processes of pasteurization, homogenization, sonication, fermentation, and microwave heating of milk significantly reduce the concentration of miRNAs (Howard et al., 2015; Golan-Gerstl et al., 2017; Zempleni et al., 2017). On the other hand, most of the milk miRNAs are encapsulated into exosomes (Benmoussa & Provost, 2019), which confer protection against degradation by temperature, RNase activity, and low pH conditions (Kosaka et al., 2010), but this information is reviewed in the “xenomiRs transported by exosomes” section. In the case of homogenization, the degradation occurs because of the forces applied in this process break the exosome membrane, releasing the miRNAs, which are now exposed to the action of RNases in milk (Howard et al., 2015; Zempleni et al., 2017). In this and next sections we will show evidence that supports the transference of milk miRNAs and others that do not.

The first study on the miRNA content of milk was performed by Izumi et al. (2012), who used microarrays and qPCR to analyze the miRNA content of colostrum and mature bovine milk. They found that colostrum contained twice the amount of miRNAs found in mature milk, and that the immune- and development-related miRNAs had significantly higher levels of expression. They also found that those miRNAs and messenger RNAs that exist naturally in milk were resistant to acidic conditions and RNAses, as well as to industrial processing conditions; this experiment is further discussed in the section about exosomes. They also suggest that further studies should be performed to confirm whether miRNAs from milk enter the infant’s gastrointestinal tract (Izumi et al., 2012).

One such study was performed by Baier et al. (2014), who analyzed three men and two women that were drinking different quantities of cow milk (0.25, 0.5, and 1.0 L) in a randomized crossover design. The authors also performed miRNA expression and depletion studies in human and murine cell cultures. The results showed significant amounts of miR-29b and miR-200c in volunteers and increased expression of runt-related transcription factor 2 (RUNX2) in blood after milk consumption.

Shu and cols. used bioinformatics to analyze 34,612 miRNAs from 194 species, including cow’s milk, breast milk, and fruits, to find those features characteristic of transportable miRNAs. They found eight groups of features that can be used to identify human circulating miRNAs and predict the likelihood of a miRNA being transferred into the bloodstream. They found that the key to miRNA transportability was the interaction between miRNAs and proteins, such as association with Ago proteins in cells. Their analysis also found 345 dietary miRNAs with a high likelihood for transfer, 73 of which are associated with exosomes. Furthermore, they validated nine bovine miRNAs, including *bta*-miR487b, *bta*-miR181b, and *bta*-miR-421 in human plasma using microRNA-sequencing analysis (Shu et al., 2015).

Laubier et al. (2015) used transgenic mice that expressed high levels of miR-30b in the milk; pups that were fed by transgenic dams did not show increased levels of miR-30b in tissues compared to pups fed by non-transgenic mice. Although high levels of miR-30b were found in the milk and stomach content of the mice, miRNAs were not significantly detected in blood (Laubier et al., 2015).

Title, Denzler & Stoffel (2015) also used genetic models to investigate miRNA uptake in mice. Newborn mice with knocked-out miR-375 and miR-200c/141 were fed by wild-type dams, but these miRNAs were not detected in the blood, intestinal epithelium, liver, and spleen tissues. Furthermore, miR-375 was barely detectable, and remained below the threshold for gene regulation. In addition, they found that milk miRNAs were swiftly degraded in the digestive tract. Altogether, their results suggest that milk-derived miRNAs do not perform a regulatory role (Title, Denzler & Stoffel, 2015). These results also contradict Baier’s results from the year before (2014) and so Zempleni’s group wrote a letter to the editor enumerating the reasons why Title’s group might have reached those conclusions. The lack of postprandial increase in miRNA levels might be attributable to “sponges”, where miRNA targets in the gastrointestinal tract and liver promote the degradation of food-derived miRNAs in a process called “first-pass elimination”—these “sponges” had been used successfully in previous experiments (Zempleni, Baier & Hirschi, 2015). Regarding Title’s group affirmation that miRNAs remained below the functional threshold, Zempleni’s group called the notion “unsound” because miRNAs can work at the femto- and picomolar range as previously described by Bryniarsky and cols. and Fabbri and cols. (Bryniarski et al., 2015; Fabbri et al., 2012). Zempleni, Baier & Hirschi (2015) agreed that miRNAs encapsulated in exosomes could contribute to the biological effects of milk. However, Title’s group argued that detection of diet-derived miRNAs is most likely the result of artifacts and over-sensitive methods, and they did not detect any evidence of uptake. And, even if the miRNA levels were undetectable, there were no changes in target gene expression in the liver, thus implying that copy numbers would not lead to gene regulation (Zempleni, Baier & Hirschi, 2015).

Auerbach et al. (2016) also obtained samples from Baier’s study, and profiled 223 small RNAs using OpenArray, and performed individual qPCR assays for some miRNAs. They also did small RNA sequencing (RNA-seq) on plasma samples from the same study. Their findings did not support Baier and cols’., results because they did not find increased levels of miR-29b or miR-200c in the plasma or PBMC three and six hours after ingestion of milk. Neither did they find miRNA alterations at these time points in a screen of 220 miRNAs, nor the miRNA-seq data show any evidence of miR-29b or miR-200c in the sequencing libraries. Furthermore, they did not find elevated levels of unique bovine miRNAs, or human/bovine homologues at the three- and nine-hour time points, as would be expected with dietary uptake. They propose several possible explanations for their results, some related to the different methodologies used, and other regarding sample quality and molecule stability that are not strong enough to justify such different results. Their results did not echo the original findings made by Baier, but agree with other recent publications on the topic, and conclude that diet-derived miRNAs are likely to serve as nutrition, but not as regulators (Auerbach et al., 2016).

In 2017, Golan-Gerstl et al., performed Next Generation Sequencing and qRT-PCR of human, goat, bovine milk and infant formula samples, to obtain their miRNA expression profiles. Interestingly, they found that infant formula contains significantly less miRNAs than human milk, and that miRNAs that were found overexpressed in human and mammalian milk were also expressed in infant formula but in a lower amount. Also, they observed that some of these miRNAs were present in exosomes and they were capable to enter to normal and tumor cells and regulate gene expression such as DNMT1. Moreover, they found that these miRNAs are associated to the immune system. These results altogether, could be related with the fact that breastfeeding infants get better protection from infections than those whose were fed with formula, through the regulation of their immune system (Golan-Gerstl et al., 2017).

Other bioinformatics studies have also failed to support transfer of miRNAs from milk to humans. Wang et al. (2018) claim to have detected bovine miRNAs (*bta*-miR-21-5p y *bta*-miR-30-5p) six hours after volunteers drank one liter of milk (Wang et al., 2018). They used RNase H2 dependent PCR, which is able to detect differences of one nucleotide between sequences; bovine and human miRNAs usually differ in two nucleotides. Wang and cols.’ study was criticized by Fromm et al. (2018), where they argue that the technique used is incapable of distinguishing human and bovine miRNAs, because miRNAs from these species are almost identical, and RNase H2 dependent PCR can only detect differences in one nucleotide if difference is in the middle of the sequence, not the ends (Fromm et al., 2018). Wang et al. (2018) suggest that when one adds equal amounts of *bta*-miR-21-5p y *hsa*-miR-21-5p, both are amplified, because bovine miRNAs amplify at 16.7 Ct, whereas human miRNAs amplify at 32.8 ct. The paper’s supplemental information, however, shows that *hsa*-miR-21-5p amplifies at 29 Ct, contrary to what is mentioned in the paper. Furthermore, the authors established a threshold of 29 Ct, and mention that any amplification above this number corresponds to human miRNAs. Notwithstanding, they report the following results for their experiment: for *bta*-miR-30a5p, Ct values were 29.7 ± 1.5 and 28.7 ± 1.8 at 0 and 6 h, respectively (*P* < 0.05). For *bta*-miR-21-5p, Ct values were 29.2 ± 3.6 and 27.0 ± 2.6 at 0 and 6 h, respectively (*P* < 0.05) (Wang et al., 2018). We observe that although the results are significantly different, the Ct values are very close to the threshold, and if we consider the standard deviation, the Ct values six hours after drinking milk are higher than the threshold that they themselves propose, and therefore these miRNAs should be considered to have human origin, not bovine. We agree with Fromm and cols that the evidence of transfer or miRNAs from milk to humans is weak, and these data should be taken with caution, and only as a starting point to explore the effectiveness of RNase H2 dependent PCR to distinguish bovine from human miRNAs.

It is difficult to conclude miRNA transfer from milk, it looks like that milk-derived miRNAs are likely to serve as nutrition, but not as regulators. The findings described are not reproducible. This variability in results is generally attributed to problems with sample quality or molecule stability. However, miRNAs are resistant to degradation and if the samples used are properly handled to preserver miRNAs, then storage shouldn’t be a big factor. On the other hand, the methodologies used have different levels of sensitivity and because of this they can produce different results. We think that it would be most appropriate to use the same sensitive methodologies in order to discern whether or not miRNAs from milk can be transferred into humans.

### XenomiRs transported by exosomes

In the previous sections, we discussed whether circulating miRNAs could be transferred through the diet, although this is not the only way that miRNAs travel. Exosomes are extracellular nanovesicles where miRNAs are packaged, shuttled, and protected from RNase activity in different biofluids like urine, saliva, plasma, serum, and breast milk; they also can shuttle DNA, RNA, proteins, and lipids from donor to recipient cells (Bahrami et al., 2018; Bayraktar, Van Roosbroeck & Calin, 2017; Rajagopal & Harikumar, 2018). These nanovesicles have a maximum diameter of 150 nm and are secreted by all type of cells (Rajagopal & Harikumar, 2018). Exosomes have gained more attention in the study of transfer of miRNAs since, through their lipid membrane, they provide xenomiRs with transportation that is resistant to degradation against harsh conditions such as RNases, freeze-thaw, and low pH, that would degrade naked miRNAs (Kosaka et al., 2010; Benmoussa & Provost, 2019). By providing protection, exosomes mediate the efficient transfer of miRNAs to their recipient cells by fusion or endocytosis (Mo et al., 2012; Boon & Vickers, 2013). It is important to note that milk miRNAs are resistant to the RNase contained in the milk by treatment with RNase and to the digestion process by mimicking the acidic environment in the gastrointestinal tract.

In 2010 Kosaka and cols., treated breast milk miRNAs with RNase, and exposed them to conditions of low pH and freeze-thaw cycles, finding that these miRNAs were resistant and stable to those treatments in comparison to synthetic miRNAs, which are degraded by these conditions. Some of these resistant breast milk miRNAs were detected in exosomes, suggesting that these vesicles protected miRNAs from degradation (Kosaka et al., 2010). Izumi et al., in 2012, found that miRNAs 155, 320, and 494 from bovine milk were stable at conditions of 37 °C, low pH and RNase treatment compared with the synthetic cel-miR-54 spiked into the sample of bovine raw milk whey, cel-miR-54 not showed stability under the condition of temperature and the presence of RNase (Izumi et al., 2012). Later in 2015 Izumi et al., performed a miRNA microarray analysis using exosomes and supernatant obtained from raw bovine milk-derived whey, they selected 20 miRNAs including miR-320 from their microarray results to be detected by qPCR and found that 18 of these miRNAs, including miR-320 (Izumi et al., 2015), which were found to be resistant to harsh conditions in their 2012 study, were present in exosomes, which could suggest that exosomes are providing protection to this miRNA in bovine raw milk.

In agreement with the aforementioned studies, most of the reports of “xenomiRs” transported by exosomes mainly focus in the exosomal miRNAs present in milk, and only one focuses on plants (Xiao et al., 2018).

Gu et al. (2012) analyzed the miRNA expression profiles in the exosomes of porcine milk. They found a high expression of immune-related miRNAs in the exosomes coming from the colostrum compared with mature milk. These miRNAs exhibited resistance to degradation when the milk was exposed to harsh conditions, similar to previous reports on meat products from Dever et al. (2015) and Link et al. (2019). Based on these observations, the exosomal miRNAs present in breast milk could be transferred to the infant body through the digestive tract (Gu et al., 2012).

In 2014, Baier and cols. did an experiment with mice and found that the activity of reporter genes decreased when they added post-prandial concentrations of milk exosomes to cell cultures. miR-29b concentrations were 61% lower in mice fed with miRNA-depleted milk, suggesting that endogenous synthesis does not compensate the miRNAs missing in the diet (Baier et al., 2014).

Lukasik & Zielenkiewicz (2014) analyzed the miRNA composition found in human and porcine breast milk exosomes looking for plant-derived miRNAs. Authors identified, in human and porcine milk, respectively, 35 and 17 miRNA species, belonging to 25 and 11 miR families. In the human samples, ath-miR166a, pab-miR951, ptc-miR472a and bdi-miR168 had the highest abundances, whereas in the porcine breast milk, zma-miR168a, zma-miR156a and ath-miR166a were the most abundant. They found that the predicted targets of these miRNAs include proteins associated with the immune system and hormone responses, and for receptors such as low-density lipoproteins, histamine and poliovirus, suggesting that these miRNAs could potentially influence human health (Lukasik & Zielenkiewicz, 2014). Plant miRNAs are easier to differentiate than mammal miRNAs, which could explain the approach taken by Lukasik and Zielenkiewicz, and the fact that the predicted targets are associated with the immune system makes sense considering that milk can confer passive immunity (Pieters et al., 2015).

Later, Bagci & Allmer (2016) repeated the analyses of Lukasik group and found that the transcripts shared by human and porcine breast milk exosome samples were highly correlated. However, they conclude that this level of correlation is extremely unlikely, and may be attributed to artifacts—and therefore, that claims of transference of plant miRNAs are likely the result of contamination (Bagci & Allmer, 2016).

Some years later, Lukasik’s group searched for five plant-derived miRNAs in the breast milk of healthy volunteers using RT-qPCR. They found miR166a, miR156a, miR157a, miR172a and miR168a in whole milk samples, but only two of them in exosomes (miR156a and miR168a). They suggest that all five miRNAs may play a role in important biological pathways of the infant such as thyroid hormone signaling, Th1 and Th2 cell differentiation, nervous system development, apoptotic processes, among others (Lukasik et al., 2017).

Moreover, it has been shown that miRNAs from bovine milk exosomes such as miR-106a, miR-451, and miR-181a could be uptaken by murine and human macrophages, affecting the function of these cells (Izumi et al., 2015; Sun et al., 2013). Regarding the mechanism by which xenomiRs contained in exosomes are transported across the intestinal mucosa and later delivered to tissues, the group of Janos Zempleni found that the intestinal uptake of miRNAs packed into bovine milk exosomes is mediated by endocytosis in humans and rats and that this process depends on the glycoproteins that the intestinal cells in human and rat present in their surface as well as on the glycoproteins localized in the surface of bovine milk exosomes (Wolf, Baier & Zempleni, 2015). Additionally, they reported that human vascular endothelial cells transport cow’s milk exosomes and their miRNA cargo by endocytosis, a process that they propose is important for the subsequent delivery to peripheral tissues (Kusuma et al., 2016).

Another in vitro and in vivo study demonstrated, that the miRNA cargo contained in milk exosomes is able to promote the proliferation of intestinal epithelial cells and the development of the digestive tract (Chen et al., 2016). Besides, it was observed that exosomes and their miRNA cargos from bovine, porcine, and murine milk across species presented a unique distribution profile and were accumulated in the brain, spleen, liver, and intestinal mucosa (Manca et al., 2018).

The study performed by Xiao and cols. does not focus in milk, instead, they looked at edible plant-derived exosome-like nanoparticles (EPDELNs), which contain miRNAs and messenger RNAs. They found that these EPDELNs can be absorbed by the gastrointestinal system, and that the miRNAs contained therein may play a role in the inflammatory response and cancer-related pathways and may regulate human messenger RNAs (Xiao et al., 2018). EPDELNs are nanoparticles that are structurally similar to exosomes, are present in edible fruit and vegetables and contain proteins, lipids, miRNAs and messenger RNAs as exosomes cargos. Also they can mediate cell–cell communications as exosomes produced by mammalian cells do (Mu et al., 2014; Zhang et al., 2016; Xiao et al., 2018)

Of the reports mentioned of the miRNAs contained in exosomes, most of them confirm transference. The evidence showed in this section as well as in the in milk-derived miRNAs section present data which shows that most of the milk miRNAs are encapsulated in exosomes and that these exosomes ensure their stability and resistance in the harsh conditions presented in milk, bloodstream, and GI tract, reinforce and support the reports with evidence of milk miRNAs transference. Altogether, represent important contributions and a promising evidence in this recent, controversial, and interesting field of research. This open an opportunity to explore more in detail the possibility of milk miRNAs transference and their activity in gene expression using more sensitivity and consensus methodologies to get more reproducible and reliable results. A possible solution for the resolution of the controversy is the labeling of molecules or exosomes, just as Wolf et al. They used fluorophore-labeled bovine milk exosomes in human colon carcinoma Caco-2 cells and rat small intestinal IEC-6 cells and showed that the uptake of bovine milk exosomes is mediated by endocytosis and depends on cell and exosome surface glycoproteins in human and rat intestinal cells. This is direct evidence of the possible mechanisms by which a human cell can absorb miRNAs present in bovine milk exosomes (Wolf, Baier & Zempleni, 2015).

On the other hand, it must be noted that the studies reviewed in this section focused on exosomes, but these are not the only carrier of miRNAs, therefore, more research is needed to study the transfer of “xenomiRs” associated to different transporters. Also, studies about exosomal miRNAs from different food sources are needed to complement and expand what we know so far regarding the interspecies transfer of miRNAs.

## Conclusions

The evidence amassed in the past years shows that plant miRNAs can enter the gastrointestinal tissue, however, there is no definitive evidence that these miRNAs pass into the bloodstream. The first evidence of miRNA transfer from the diet was published eight years ago (Zhang et al., 2012a). Since then, different research groups have tried to replicate these findings, using both plant and animal sources. Some groups have claimed success, whereas others claim that there is no transfer of miRNAs. In spite of the biological barriers as well as the mechanisms of cellular uptake that may limit miRNA transference, several diet-derived miRNAs can transfer into the circulating system and target human and porcine genes for transcription regulation genes, which adds arguments that miRNAs can be absorbed from the diet and target specific genes by regulating their expression. However, many other studies show that cross-kingdom transfer of exogenous miRNAs appears to be insignificant and not biologically relevant. The main source of controversy in plant studies is the lack of reproducibility of the findings. One of the most promising results is the finding that plant miRNAs in beebread regulate honeybee caste development, and cause similar changes when fed to *Drosophila* (Zhu et al., 2017). These results suggest that plant-derived miRNAs play an important role in the regulation of animal genes, however other studies have attempted to demonstrate the transfer of plant miRNA to bees with negative results (Snow et al., 2013; Masood et al., 2016).

Regarding bovine miRNAs, the studies concluded that miRNAs can survive the cooking process of meat (Dever et al., 2015), nevertheless our results shows that these bovine miRNAs are not transferred to human bloodstream (Mata-Cardona, 2016). More controlled studies are needed that can endorse or reject the transfer of miRNAs from meat to humans. The only evidence pointing to the possible transfer is the finding that some foods of animal origin are ubiquitously present in feces and normal mucosa but further studies are needed to evaluate the functional interaction between diet-derived miRNAs and GI tract (Link et al., 2019).

Most of the reports of the miRNAs contained in exosomes confirm the miRNA transference. miRNAs encapsulated in exosomes ensure their stability and resistance in the harsh conditions presented in milk, bloodstream, and GI tract, reinforce and support the reports with evidence of milk miRNAs transference. Further studies should be performed to analyze the transfer of miRNAs by exosomes or other transporters such as lipoproteins and Ago-2. It is also important to elucidate the biological function of “xenomiRs” and determine whether they could be involved in the regulation of gene expression and other processes in the consuming organism.

We note that the main source of controversy is the fact that all the studies that provide evidence of transfer have produced conflicting results when replicated, with several authors indicating that are sample contamination or artifacts of sequencing (Bagci & Allmer, 2016; Kang et al., 2017). Regardless of the model organism, the source of miRNAs, or the approach—bioinformatics or in vivo—the issue of transfer of miRNAs from the diet remains in doubt.

Another interesting aspect that we have observed is that the most important techniques for the quantification of nucleic acids, such as, real-time PCR, microarrays and deep sequencing have not been enough to discern the transfer of miRNAs, perhaps it is time to start to try new techniques. In recent years, several authors have begun to describe new tools for the specific detection of miRNAs. A technique that would be useful is to label microRNAs for subsequent detection. In this sense, new protocols have begun to be described that could work to make preliminary studies in laboratory animals (Croci et al., 2019).

Our understanding of the cross-kingdom talk of miRNAs is still in infancy, and it is too early to make a final statement on whether microRNAs are transferred, much less if they perform any regulatory role. We hope that the following years bring evidence that will settle the debate on this recent, controversial, and interesting field of research.

## Additional Information and Declarations

### Data Availability

The following information was supplied regarding data availability: This is a literature review and did not generate raw data.
